# Placenta Accreta Spectrum: Diagnostic Challenges and Management Strategies

**DOI:** 10.3390/diagnostics16050760

**Published:** 2026-03-03

**Authors:** Zlatko Kirovakov, Angel Yordanov, Eva Tsoneva

**Affiliations:** 1 Faculty for Public Health and Healthcare, Burgas State University Prof. Dr. Assen Zlatarov, 8010 Burgas, Bulgaria; kirovakov@yahoo.com; 2 Department of Gynecologic Oncology, Medical University Pleven, 5800 Pleven, Bulgaria; 3 Department of Reproductive Medicine, Specialized Hospital for Active Treatment of Obstetrics and Gynaecology “Dr Shterev”, 1330 Sofia, Bulgaria; dretsoneva@gmail.com

**Keywords:** placenta previa, antenatal diagnosis, cesarean hysterectomy, conservative management, multidisciplinary care, obstetric hemorrhage

## Abstract

This narrative review presents an updated overview of the etiology, pathophysiology, diagnostic approaches, and management strategies for Placenta Accreta Spectrum (PAS), with emphasis on clinical implications and current gaps in evidence. PAS is associated with substantial maternal morbidity and mortality, with reported maternal mortality rates approaching 7%. Affected patients often experience prolonged hospitalization, repeated surgical interventions, and long-term psychological and emotional consequences. The development of PAS is primarily attributed to impaired decidualization in areas of uterine scarring, resulting in abnormal adherence or invasion of chorionic villi into the myometrium. Optimal outcomes in high-risk pregnancies depend on early antenatal identification using characteristic pathological and imaging findings. Current evidence supports planned cesarean hysterectomy as the safest and most definitive treatment for most patients, whereas conservative and uterus-preserving approaches should be reserved for carefully selected cases managed in specialized centers. Further progress in PAS management requires standardized diagnostic criteria, prospective evaluation of conservative strategies, and improved access to multidisciplinary expertise.

## 1. Introduction

Placenta Accreta Spectrum (PAS) encompasses a group of placental implantation disorders characterized by abnormal adherence of the placenta to the uterine wall, ranging from superficial attachment to deep myometrial invasion or penetration beyond the uterine serosa [1,2]. This spectrum includes placenta accreta, increta, and percreta, representing increasing depths of trophoblastic invasion into the myometrium. Clinically, PAS is associated with a continuum of pathological severity and poses a major risk for severe obstetric hemorrhage, particularly during attempted placental separation at delivery [1,3,4]. Although PAS remains relatively uncommon, it carries significant maternal and neonatal risks, including massive hemorrhage, infection, urinary tract injury, fistula formation, and increased neonatal morbidity and mortality [5,6]. Maternal mortality associated with PAS varies widely depending on geographic region, healthcare resources, case severity, and centralization of care. Earlier reports from non-centralized settings described mortality rates approaching 7% [7], whereas contemporary series from specialized centers report substantially lower rates [3,8]. The growing incidence of PAS, together with the high proportion of cases diagnosed intrapartum, underscores the importance of heightened clinical vigilance, particularly in patients with known risk factors and chronic antenatal bleeding.

Over the same period during which cesarean delivery rates have risen worldwide, a parallel increase in abnormal placentation—particularly Placenta Accreta Spectrum (PAS)—has been observed. Contemporary epidemiological data demonstrate marked regional variability in cesarean section rates, ranging from approximately 5% in sub-Saharan Africa to over 40% in Latin America and the Caribbean, with intermediate but steadily rising rates in Europe (≈25%) and Asia (≈23%) [9,10]. Importantly, PAS incidence closely mirrors these regional patterns, with the highest reported prevalence occurring in settings characterized by long-standing high cesarean utilization and a growing population of women with uterine scars [1,11].

Although precise population-level PAS incidence remains difficult to quantify due to heterogeneity in diagnostic criteria and reporting practices, multiple cohort studies and systematic reviews consistently demonstrate a strong dose–response relationship between the number of prior cesarean deliveries and the risk of PAS, particularly when combined with placenta previa [3,4,12,13]. In regions where elective and repeat cesarean sections predominate, the expanding cohort of women with scarred uteri creates the epidemiological conditions for a proportional increase in PAS incidence in subsequent pregnancies. Based on this well-established association, it is reasonable to predict that continued growth in cesarean delivery rates—especially non-medically indicated primary and repeat procedures—will be accompanied by a further rise in PAS cases over the coming decades [9,11].

These trends support the characterization of PAS as a largely iatrogenic and potentially preventable obstetric disorder, underscoring the importance of public health strategies aimed at reducing unnecessary primary cesarean sections and limiting cascades of repeat surgical deliveries [1,4,10].

The term placenta accreta was first clearly characterized by Irving and Hertig in 1937, who described failure of placental separation due to abnormal adherence of chorionic villi directly to the myometrium in the absence of the decidua basalis and Nitabuch’s layer [14]. Over time, clinicians recognized that abnormal placental attachment exists on a spectrum, depending on the depth of trophoblastic invasion. Subsequent observations revealed that abnormal placentation occurs along a pathological continuum rather than as a single entity. This understanding led to the adoption of the term Placenta Accreta Spectrum, which integrates superficial adherence (accreta) [15], deep myometrial invasion (increta) [16], and transmural invasion involving surrounding pelvic structures (percreta) [17].

Historically, PAS was classified by depth of invasion [18]:

1. Accreta (also called “creta” or “vera/adherenta”): attachment to myometrium without invasion;
2. Increta: villi invade into the myometrium;
3. Percreta: villi penetrate through the myometrium and serosa, sometimes into adjacent structures.

In 2019, FIGO introduced a formal clinical classification, refining these entities as [15]:

● Grade 1: abnormally adherent placenta;
● Grade 2: abnormally invasive placenta (increta);
● Grade 3: abnormally invasive placenta (percreta).

The incidence of PAS is rising globally, largely due to increasing cesarean delivery rates, with transfusion at delivery occurring at up to half of the patients [3], intensive care unit (ICU) admission after delivery is common, and operative injury to the urinary tract occurs in 5–30% of cases [19]. Patients with PAS have a lower quality of life, longer-lasting psychological and emotional issues, more frequent hospital stays, and the need for additional procedures [20,21]. By pooled estimates, PAS now occurs in 0.17% (1 in 588) of pregnancies (95% CI 0.01% to 1.1%) [12], as compared with the much lower incidence in the 1970s and 1980s at 0.02% (1 in 4027) and 0.04% (1 in 2510), respectively [3,4]. The high incidence rates of placenta accreta could be attributed to a change in risk factors, most notably the increased rate of cesarean delivery [1,2,9,10]. Considering the rising incidences and the obstetric implications of PAS-related disorders, the present review provides a comprehensive analysis of the etiology and pathophysiology of the condition, diagnosis and the management strategies for patients, as well as the clinical implications and applications to fill the existing research gap on the topic.

### Aim of the Review

The primary aim of this narrative review is to critically synthesize current evidence on the etiology, pathophysiology, diagnostic modalities, and management strategies of Placenta Accreta Spectrum (PAS), with a particular focus on diagnostic challenges and real-world clinical decision-making. Specifically, this review seeks to address the following questions:

(1) What are the current limitations and strengths of antenatal diagnostic tools for PAS?
(2) How should diagnostic findings guide individualized management strategies?
(3) What gaps remain in conservative management and future risk stratification?

By integrating recent advances with clinical algorithms, this review aims to support clinicians in optimizing maternal outcomes while identifying priorities for future research.

## 2. Methodology of Literature Selection

This narrative review was based on a targeted, non-systematic search of the medical literature focusing on Placenta Accreta Spectrum disorders. Electronic searches were performed primarily using PubMed/MEDLINE, supplemented by manual screening of reference lists from key review articles and international consensus statements. Search terms included combinations of Medical Subject Headings (MeSH) and free-text keywords such as “placenta accreta spectrum,” “placenta increta,” “placenta percreta,” “antenatal diagnosis,” “ultrasound,” “magnetic resonance imaging,” “cesarean hysterectomy,” and “conservative management.”

Priority was given to recent meta-analyses, prospective cohort studies, international guidelines, and large multicenter observational studies published in English. Case reports were included selectively when addressing rare presentations or specific management challenges. Given the narrative nature of this review, no formal systematic review protocol or quantitative synthesis was undertaken.

## 3. Etiology and Pathophysiology

A clear understanding of the etiological mechanisms underlying PAS is essential for effective risk stratification, early diagnosis, and informed management planning. The predominant pathogenic mechanism is believed to involve defective decidualization at sites of previous uterine injury, allowing abnormal placental anchoring or direct trophoblastic invasion into the myometrium. Areas of scarring within the uterine wall create a permissive environment for aberrant placental attachment, bypassing the normal decidual barrier [10,11].

Several well-established risk factors have been associated with PAS, including placenta previa, advanced maternal age, conception following in vitro fertilization, smoking, and prior uterine surgery. Among these, previous cesarean delivery represents the most significant contributor to PAS risk, particularly when combined with placenta previa [12,22]. Additional intrauterine procedures, such as curettage or endometrial ablation, further increase susceptibility by disrupting the integrity of the endometrial lining [10]. The coexistence of placenta previa and PAS markedly elevates the risk of severe peripartum hemorrhage and contributes to complex postoperative courses characterized by high maternal morbidity [4,13]. Importantly, the absence of definitive sonographic findings does not exclude PAS, emphasizing the need for comprehensive clinical risk assessment alongside imaging.

The effective management of high-risk PAS pregnancies requires the recognition of prepartum signs through pathological findings indicative of the condition. From a pathophysiological perspective, PAS is characterized by failure of normal decidual formation and loss of the Nitabuch layer, enabling uncontrolled trophoblastic invasion into the myometrium [4,23,24]. However, the pathogenesis is multifactorial and incompletely understood, involving a combination of decidual defects, aberrant angiogenesis, altered growth factor signaling, and immune dysregulation at the maternal–fetal interface. These processes collectively compromise placental boundary regulation and facilitate invasive implantation [1]. The primarily confirmed pathological processes include decidual defects, abnormal placental attachment, and dysregulated angiogenesis and growth factors [25,26], all of which can compromise the decidua, leading to abnormal placental invasion into the myometrium [1]. The pathological sonographic findings indicative of PAS includes intraplacental lacunae, loss of the hypoechogenic retroplacental (clear) zone, an irregular barrier between the uterus and bladder, interruption between the uterus and bladder (bright bladder line), or exophytic tumors within the bladder [10]. In advanced cases, interpretation of pathological specimens may be challenging due to extreme thinning of the residual myometrium [1]. Elucidating these mechanisms remains critical for improving diagnostic accuracy and refining therapeutic strategies.

## 4. Diagnosis

Accurate prenatal identification of PAS is fundamental to optimizing delivery planning and improving maternal outcomes. Histopathological confirmation remains the diagnostic gold standard; however, definitive assessment is typically possible only after surgical intervention, as placental sampling is limited to hysterectomy or partial uterine resection specimens [1]. A complete assessment of placental implantation can only be carried out following surgical removal since placental pathology sampling is usually limited to specimens from uterine excision or partial resection [27,28,29].

Ultrasound serves as the primary modality for antenatal screening and diagnosis of PAS due to its accessibility, safety profile, and real-time assessment capabilities (Table 1). Key sonographic indicators include loss of the retroplacental clear zone, irregular placental lacunae, abnormal myometrial thinning, disruption of the bladder–uterine interface, and turbulent vascular flow on color Doppler imaging [30,31,32]. In high-risk populations, ultrasound demonstrates sensitivity approaching 90%, particularly when standardized criteria and advanced techniques are employed [33].

The integration of 2D grayscale ultrasound, color Doppler, and 3D ultrasound enables an accurate assessment of the type and extent of placental invasion which offer crucial data that facilitates customized planning and management, allowing for the prevention of potential issues and enhancing the outcomes for mothers and newborns [34]. Ultrasound examination during the second and third trimesters is crucial in screening for PAS due to the clear manifestation of indicative features of invasive implantation and 3D ultrasound provides a detailed representation of the placental structure and its relationship with the uterine wall for assessment of complex pathophysiological manifestations such as posterior placental invasion [15,33,35].

**Table 1:** Old vs. New Ultrasound Criteria for Diagnosing Placenta Accreta Spectrum (PAS).

Feature	Old/Classical Criteria	New/Standardized Criteria	References
Clear zone	Subjective loss or thinning of retroplacental hypoechoic zone	Objective loss/thinning < 1 mm of clear zone	Hessami et al., 2024 [34]; Jauniaux et al., 2022 [36]
Placental lacunae	Few or nonspecific placental lakes	Numerous irregular lacunae with turbulent Doppler flow (“Swiss cheese”)	Self et al., 2025 [29]; Jauniaux et al., 2020 [33]
Myometrial thickness	Not routinely measured; vague descriptors	Myometrial thinning (<1 mm) or absence at placental bed	Hessami et al., 2024 [34]; Arakaza et al., 2023 [15]
Bladder interface	Loss of echogenic interface (non-specific)	Disruption of uterine serosa–bladder interface	Patel-Lippmann et al., 2023 [17]; Tinari et al., 2021 [37]
Placental contour	Not evaluated	Placental bulge deforming uterine outline	Thiravit et al., 2021 [35]; Self et al., 2025 [29]
Bridging vessels	Not identified or assessed	Vessels traversing from placenta into bladder/serosa	Patel-Lippmann et al., 2023 [17]; Self et al., 2025 [29]
Color Doppler use	Rarely used or inconsistent	Essential: subplacental hypervascularity, chaotic flow	Mohamed et al., 2025 [31]; Gulati et al., 2021 [30]
Exophytic masses	Not described	Focal mass protruding beyond serosal surface	Patel-Lippmann et al., 2023 [17]
Assessment of invasion	Inconsistent, subjective	Integrated grayscale and Doppler assessment	Hessami et al., 2024 [34]; Jauniaux et al., 2022 [36]
Reproducibility	Low; highly operator-dependent	Improved with standardized training	Jauniaux et al., 2022; [36] Bonanni et al., 2025 [38]
Sensitivity/Specificity	Variable: 60–80% sensitivity	Higher sensitivity (~90%) and specificity (80–95%) in high-risk cases	Hessami et al., 2024 [34]; Sentilhes et al., 2022 [39]

Sensitivity and specificity ranges refer to pooled estimates from meta-analyses and large cohort studies conducted in high-risk populations (e.g., placenta previa with prior cesarean delivery) using standardized ultrasound criteria, primarily derived from Hessami et al. [34]. and Jauniaux et al. [36]. Diagnostic performance may be lower in unselected or low-risk populations.

The application of Magnetic Resonance Imaging (MRI) in PAS diagnosis has gained prominence due to its capacity to give comprehensive anatomical information, especially when ultrasound results are ambiguous or constrained by technical limitations or inconclusive. Although ultrasound is effective for early screening, its accuracy may be limited by maternal obesity, the posterior placement of the placenta, or excessive intestinal gas, which could obscure crucial imaging information [32,37,40,41]. MRI, on the other hand, provides excellent soft tissue contrast and high-resolution imaging, which makes it a vital tool for determining the extent of placental invasion, myometrial thinning, and the involvement of nearby organs [1]. Also, MRI is especially helpful in more complicated circumstances where precise preoperative planning is necessary due to the more complex anatomical linkages, such as placenta percreta or multiple gestations [42,43,44]. MRI’s diagnostic value varies depending on the stage of pregnancy, with unique imaging characteristics developing as the placenta grows [17,45,46,47]. MRI is especially useful in early pregnancy (11–14 weeks) for detecting early indicators of placental invasion, such as aberrant vascular signals and the loss of the placenta–myometrium barrier [1]. MRI’s capacity to show the depth and extent of placental invasion increases as pregnancy moves into the second and third trimesters, offering critical data for risk assessment and surgical planning. A comparative understanding of the diagnostic roles, advantages, and limitations of ultrasound, magnetic resonance imaging (MRI), and histopathological examination is essential for accurate diagnosis and optimal management of PAS. While ultrasound remains the first-line modality, MRI serves as an adjunct in anatomically challenging or inconclusive cases. Histopathology, though considered the gold standard, is only confirmatory postoperatively. The following table summarizes the key features of each modality as they relate to the evaluation of PAS (Table 2).

**Figure 1:**
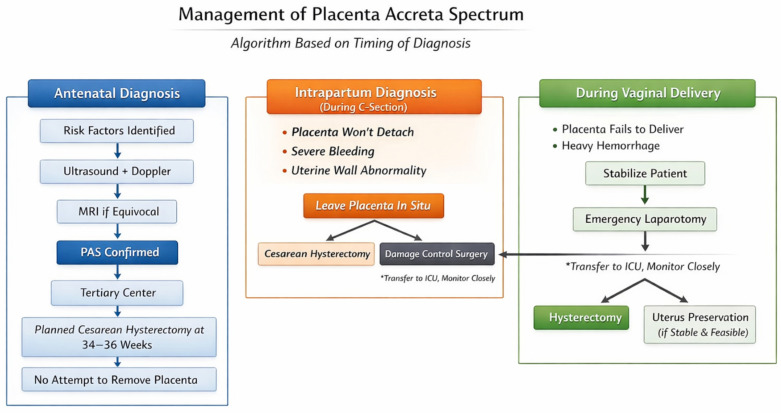
Clinical algorithm for the management of Placenta Accreta Spectrum (PAS) according to the timing of diagnosis. * Refers to transfer to ICU.

While existing guidelines outline general principles, practical, scenario-based clinical algorithms are limited in the literature. As a novel contribution of this review, we propose a structured clinical algorithm based on the timing of PAS diagnosis-antenatally, intraoperatively during cesarean delivery, or postpartum during vaginal delivery. This algorithm is designed to aid clinicians in real-time decision-making, integrating current evidence with clinical pragmatism and resource availability (Figure 1).

Rationale and Evidence Basis for the Proposed Clinical Algorithm

The proposed clinical algorithm is derived from existing international consensus statements, prospective cohort data, and large multi-institutional database studies rather than intended as a replacement for formal guidelines. It integrates recommendations from FIGO, the International Society for Placenta Accreta Spectrum (IS-PAS), and the PACCRETA prospective study, which emphasize early antenatal diagnosis, avoidance of placental removal, planned cesarean hysterectomy in specialized centers, and cautious selection of conservative management strategies.

The algorithm specifically addresses areas where existing guidelines provide general principles but lack structured, scenario-based decision pathways, particularly in cases of unexpected intraoperative or postpartum diagnosis. Evidence supporting key decision points is primarily based on observational studies and expert consensus, reflecting the ethical and practical limitations of randomized trials in PAS management.

This algorithm outlines stepwise management pathways based on whether PAS is suspected antenatally, diagnosed unexpectedly during cesarean delivery, or identified postpartum following vaginal delivery. It emphasizes avoidance of placental removal, early multidisciplinary involvement, escalation to cesarean hysterectomy when indicated, and strict criteria for conservative management. The algorithm is intended as a pragmatic decision-support tool adaptable to institutional resources and clinical expertise. It assumes availability of a multidisciplinary team, access to blood products, anesthesia expertise, and postoperative intensive care support. Conservative strategies are applicable only in selected, hemodynamically stable patients managed in specialized centers. The algorithm may not be applicable in low-resource settings, emergency scenarios without surgical backup, or institutions without PAS experience. Also, it is intended as a flexible clinical framework and may require modification based on institutional resources, emerging evidence, and individual patient factors; prospective validation studies are required to assess its clinical impact and limitations.

- Pas Suspected or Diagnosed Antenatally

In cases where PAS is suspected during routine antenatal care, screening should begin with a detailed obstetric history focusing on known risk factors such as prior cesarean section, placenta previa, uterine surgeries, or in vitro fertilization [13,34]. First-line imaging involves a combination of transabdominal and transvaginal ultrasound, supplemented by color Doppler to evaluate placental vascularity, myometrial integrity, and bladder interface [29,33]. When sonographic findings are inconclusive-particularly in patients with posterior placenta or high BMI-magnetic resonance imaging (MRI) should be employed to clarify the diagnosis and assess the depth and topography of invasion [17,47].

Once PAS is confirmed, patients should be referred to a tertiary center with a dedicated multidisciplinary team, including maternal–fetal medicine specialists, anesthesiologists, neonatologists, urologists, and interventional radiologists [38,48]. Delivery should be scheduled between 34 and 36 weeks of gestation to optimize fetal maturity while minimizing the risk of spontaneous bleeding [36]. A planned cesarean hysterectomy should be performed with the placenta left in situ, avoiding any attempt at placental removal due to the high risk of massive hemorrhage [19,49]. Preoperative preparation must include arrangement for adequate blood products, availability of surgical subspecialties, and ICU support [39].

B. Unexpected Pas Diagnosed During Cesarean Delivery

Intraoperative diagnosis of PAS may occur unexpectedly when the placenta fails to detach, there is profuse bleeding, or the uterine wall appears abnormally thin or distorted [3,4]. In these cases, immediate steps must be taken to minimize maternal risk. Manual removal of the placenta should be strictly avoided, as this can precipitate catastrophic hemorrhage. Instead, the placenta should be left in situ, and senior surgical support must be summoned promptly [38]. Depending on the clinical stability of the patient and the experience of the surgical team, the procedure should either proceed to a cesarean hysterectomy or adopt a temporary damage control approach [5].

Postoperative management should include transfer to a high-dependency or intensive care unit. If a conservative approach was initially chosen due to hemodynamic instability or surgical constraints, patients must be closely monitored for delayed hemorrhage or infection, and a plan for staged definitive surgery should be in place [39,49].

C. Pas Suspected During Vaginal Delivery

PAS may also present in the context of vaginal delivery, typically when the placenta fails to separate spontaneously or when unexpected hemorrhage occurs. In such cases, repeated attempts at manual removal should be avoided, and PAS should be immediately suspected [33]. Bedside ultrasound may assist in confirming the diagnosis, but clinical judgment remains paramount. Stabilization of the patient’s hemodynamic status takes priority, followed by rapid preparation for emergency laparotomy [33].

If PAS is confirmed intraoperatively, the surgical team must decide between hysterectomy and uterine-conserving approaches based on the extent of invasion, bleeding severity, and availability of expertise. Uterine preservation should only be attempted in highly selected cases with stable patients, minimal invasion, and immediate access to multidisciplinary surgical support [39,50,51].

## 5. Treatment

Diagnostic findings directly influence management decisions, including timing of delivery, referral to specialized centers, and selection between definitive surgical and conservative approaches. Therefore, treatment strategies for PAS must be interpreted in the context of antenatal imaging, clinical risk factors, and institutional expertise.

Effective management of PAS relies more on institutional preparedness and multidisciplinary coordination than on the expertise of any single clinician [2]. Planned cesarean hysterectomy remains the cornerstone of treatment for most patients, offering the highest likelihood of maternal safety. Nevertheless, uterus-preserving strategies may be considered in carefully selected cases under strict clinical criteria. Regardless of the chosen approach, successful outcomes depend on early diagnosis, meticulous preoperative planning, and the availability of specialized surgical, anesthetic, and interventional resources. Reported success rates for conservative management vary widely depending on outcome definitions, follow-up duration, and patient selection criteria, limiting direct comparison between studies.

### 5.1. Surgical Management: Planned Cesarean Hysterectomy

Planned cesarean hysterectomy with the placenta left in situ is currently the most definitive and evidence-supported treatment for confirmed PAS, particularly in patients who have completed childbearing. It is endorsed by major international guidelines, including FIGO and IS-PAS [36,38]. The key principle is to avoid manual removal of the placenta, which can provoke uncontrollable hemorrhage due to the absence of a normal cleavage plane between the placenta and myometrium.

The procedure should be performed electively between 34 and 36 weeks of gestation, ideally before the onset of labor or significant vaginal bleeding. Preoperative planning involves coordination among maternal–fetal medicine specialists, anesthesiologists, urologists, interventional radiologists, and neonatologists. Blood products must be prepared in advance, and the operating team should be experienced in managing complex pelvic surgery.

Intraoperatively, the incision should be made away from the placental location, often via a classical or high transverse uterine incision. Following fetal delivery, the uterus is removed en bloc with the placenta left untouched. Intraoperative complications may include massive hemorrhage, bladder injury, or ureteric damage, especially in cases of percreta with anterior placental location [48]. Bladder dissection may be required when the placenta invades the uterovesical space. Postoperative care should include ICU monitoring, thromboprophylaxis, and psychological support due to the high rate of post-surgical morbidity [21].

### 5.2. Conservative Management Approaches

Conservative management of placenta accreta spectrum offers an alternative to cesarean hysterectomy in selected patients who desire future fertility, have focal invasion, or are poor surgical candidates due to comorbidities or extensive pelvic involvement. These approaches aim to preserve the uterus while minimizing surgical trauma and maternal morbidity. According to current literature, the principal conservative strategies include expectant management, segmental resection, the Triple-P procedure, and adjunctive therapies such as methotrexate and uterine artery embolization [40,52] (Table 3). It is important to emphasize that reported uterine preservation rates approaching 78–80% reflect highly selected patient populations managed in specialized centers, with success typically defined as avoidance of immediate hysterectomy rather than long-term reproductive or obstetric outcomes.

#### 5.2.1. Expectant Management

Expectant management involves leaving the placenta in situ after fetal delivery without any attempt at manual separation. This strategy relies on gradual placental resorption over weeks to months and requires intensive postpartum surveillance. Success rates of up to 78% have been reported, though serious risks include delayed hemorrhage, sepsis, uterine necrosis, and need for emergency hysterectomy [52]. Patients must be closely monitored with serial ultrasound (often Doppler-enhanced) and β-hCG levels to track placental involution. Long-term follow-up is essential.

#### 5.2.2. Methotrexate Therapy

Methotrexate has been used as an adjunct to expectant management to accelerate placental involution by targeting trophoblastic tissue. However, evidence for its efficacy is limited and inconsistent. Some small series suggest shorter time to placental resorption and reduced need for secondary procedures, while others report no significant benefit [51]. Its use is further complicated by risks of hepatotoxicity, myelosuppression, and mucositis. Current guidelines do not recommend routine methotrexate use, but it may be selectively considered on a case-by-case basis, particularly where close follow-up is possible.

#### 5.2.3. Segmental Resection (One-Step Conservative Surgery)

This technique involves localized resection of the placenta along with the invaded myometrium, followed by uterine wall reconstruction. It is most appropriate for focal PAS with clear margins of invasion and requires experienced surgical teams. Compared to hysterectomy, this approach preserves fertility but carries risks of hemorrhage, uterine rupture in future pregnancies, and need for reoperation if resection is incomplete [39].

#### 5.2.4. Triple-P Procedure

The Triple-P strategy—comprising perioperative placental localization, pelvic devascularization, and placental–myometrial excision—is a more structured conservative approach. Following localization of the superior placental edge via ultrasound, preoperative internal iliac or uterine artery balloons are used to devascularize the uterus. The invaded myometrium and placenta are then excised en bloc, and the uterus is reconstructed. This technique reduces blood loss and improves uterine preservation rates, but requires interventional radiology and surgical expertise not available in all centers [52].

#### 5.2.5. Adjunctive Interventions

Adjunct measures such as uterine artery embolization (UAE), temporary internal iliac balloon occlusion, and ligation of uterine or hypogastric arteries can be combined with conservative strategies to reduce blood loss. UAE is often used postpartum in expectant management or after failed resection to control secondary hemorrhage. While these methods do not replace surgical treatment, they are valuable tools when immediate bleeding control is necessary [51].

### 5.3. Long-Term Maternal and Reproductive Outcomes After Placenta Accreta Spectrum

Long-term outcomes following Placenta Accreta Spectrum (PAS) extend beyond the immediate peripartum period and remain incompletely characterized. Women undergoing cesarean hysterectomy experience permanent loss of fertility and are at increased risk of long-term physical and psychological sequelae, including chronic pelvic pain, sexual dysfunction, and impaired quality of life [20,21]. Patient-reported outcome studies indicate higher rates of anxiety, depressive symptoms, and post-traumatic stress disorder following PAS-related hysterectomy, particularly in cases complicated by massive hemorrhage or intensive care admission [20,21].

In patients managed conservatively, preservation of the uterus does not equate to absence of long-term morbidity. Delayed postpartum hemorrhage, infection, uterine necrosis, and the need for secondary hysterectomy have been reported weeks to months after delivery [39,50,52]. Moreover, subsequent pregnancies following conservative management are associated with a substantial risk of recurrent PAS, placenta previa, uterine rupture, and severe obstetric hemorrhage, necessitating early referral and intensive surveillance [39,52,54]. Reported recurrence rates vary widely across studies, reflecting heterogeneity in patient selection, depth of placental invasion, and duration of follow-up.

Importantly, the existing literature on long-term reproductive outcomes after PAS is limited by small sample sizes, retrospective designs, and inconsistent outcome definitions. While successful subsequent pregnancies have been described, they remain high-risk and are frequently complicated by repeat abnormal placentation or preterm delivery [23,52]. These uncertainties highlight the need for prospective, long-term follow-up studies evaluating not only reproductive outcomes but also psychological well-being and quality of life after both definitive and conservative PAS management strategies [20,23].

## 6. Clinical Implications and Applications

Comprehensive management of PAS requires individualized risk assessment to guide clinical decision-making, resource allocation, and patient counseling. Risk stratification should incorporate obstetric history, imaging findings, and relevant clinical parameters. Early antenatal diagnosis facilitates timely referral to specialized centers, coordination of multidisciplinary teams, and preparation for potential massive transfusion [55,56].

For most patients, delivery planning involves scheduled cesarean hysterectomy between 34 and 36 weeks of gestation, prior to labor onset or spontaneous hemorrhage. This approach allows optimal anesthetic management, surgical control, and neonatal preparedness [57]. Conservative and fertility-preserving strategies occupy a limited but important role and should be reserved for highly selected patients with localized disease, strong reproductive wishes, and access to advanced multidisciplinary care.

Uterus-preserving techniques should be reserved for carefully selected patients, often those with localized disease and a strong desire for future reproduction, and only in facilities with considerable PAS expertise [4,54]. Ultimately, favorable maternal outcomes are achieved through early recognition, structured imaging pathways, coordinated multidisciplinary planning, and alignment of management strategies with disease severity, patient preferences, and institutional capabilities.

## 7. Future Perspectives: Placental Biomarkers and Prognostic Stratification

Emerging evidence suggests that placental histopathology and molecular profiling may provide valuable insights into the pathogenesis, severity, and prognostic stratification of Placenta Accreta Spectrum (PAS). Abnormal expression of markers involved in trophoblast invasion, angiogenesis, extracellular matrix remodeling, and immune regulation has been increasingly reported in invasive placentation. In particular, alterations in vascular endothelial growth factor signaling, matrix metalloproteinases, and decidualization-related pathways appear to play a central role in abnormal placental adherence and invasion [14].

Molecular and immunohistochemical studies have demonstrated that PAS is associated with disrupted placental–uterine interface biology, including excessive trophoblastic infiltration and dysregulated angiogenesis, which may correlate with clinical severity and surgical complexity [58,59]. Furthermore, pathological investigations highlight that impaired decidual formation and abnormal remodeling of the maternal–fetal interface are key drivers of invasive placentation, suggesting that tissue-based biomarkers could potentially aid in risk stratification and prediction of adverse outcomes [60].

Although these findings are promising, the clinical applicability of placental biomarkers remains limited by heterogeneity in study design, small sample sizes, and lack of standardized assessment protocols. At present, biomarker evaluation is primarily restricted to post-delivery histopathological analysis and has no established role in routine prenatal diagnosis. Future prospective studies integrating immunohistochemical markers with imaging findings and clinical risk factors are required to validate their prognostic value and to determine whether biomarker-based approaches could contribute to earlier diagnosis, individualized management strategies, or selection of candidates for conservative treatment.

## 8. Limitations and Gaps in Research

Despite advances in diagnostic techniques and surgical management, significant limitations persist in the evidence base surrounding PAS. Diagnostic uncertainty remains a major challenge, as both ultrasound and MRI may struggle to reliably distinguish between degrees of placental invasion. Variability in imaging interpretation, operator expertise, and placental location can result in under- or overestimation of disease severity, directly impacting surgical planning and patient counseling.

Additionally, long-term outcomes of conservative management remain poorly defined due to the absence of standardized monitoring protocols and heterogeneity in reported outcomes. Resource constraints further limit the applicability of guideline-recommended care, as access to multidisciplinary teams and advanced imaging or transfusion services is not universally available. Addressing these gaps will require standardized diagnostic frameworks, prospective studies evaluating conservative strategies, and broader access to specialized care, particularly in low-resource settings. As a narrative review, this work is inherently limited by potential selection bias and the absence of formal quantitative synthesis, which may affect the generalizability of some conclusions.

## 9. Conclusions and Recommended Next Steps

Placenta Accreta Spectrum represents a continuum of abnormal placental implantation disorders associated with substantial maternal morbidity and mortality, primarily due to severe obstetric hemorrhage. Advancing the care of affected patients necessitates continued refinement of diagnostic strategies, development of structured clinical pathways, and expansion of multidisciplinary referral networks. Future research should focus on defining safe and effective conservative management protocols, evaluating long-term reproductive outcomes, and identifying early biomarkers of invasive placentation. Women with a history of uterine surgery should receive targeted counseling regarding recurrence risk in subsequent pregnancies. Ultimately, early risk stratification, coordinated multidisciplinary care, and standardized imaging assessment remain central to improving outcomes in PAS.

## Figures and Tables

**Table 2:** Comparison Table: Ultrasound vs. MRI vs. Histopathology in PAS Diagnosis.

Feature	Ultrasound	MRI	Histopathology
Role	First-line screening and diagnosis	Adjunctive imaging for complex cases	Definitive confirmation (post-surgical)
Sensitivity	77–93%	80–89%	Reference standard/postoperative confirmation
Specificity	71–96%	70–87%	Reference standard/postoperative confirmation
Best for	Real-time dynamic imaging, anterior placenta	Posterior placenta, lateral extension, bladder invasion	Exact depth and tissue involvement
Limitations	Operator-dependent; limited by BMI/posterior placenta	Cost, limited availability, radiologist expertise	Not usable for prenatal planning
Accessibility	Widely available, cost-effective	Limited to specialized centers	Postpartum only
Other Notes	Combines well with color Doppler and 3D	Valuable for surgical mapping and parametrial invasion	Required for staging and research histology

**Table 3:** Conservative Management Strategies for Placenta Accreta Spectrum (PAS): Methods, Success Rates, and Selection Criteria.

Method	Type	Success Rate	Patient Selection Criteria	Key References
Expectant management (placenta left in situ)	Non-surgical	~78–80% uterine preservation	Hemodynamically stable No active hemorrhage or infection Limited invasion (accreta/increta) High compliance with follow-up	[36,39,52]
Uterine artery embolization (UAE)	Minimally invasive	~70–80% (varies by series)	Stable patient Focal disease Access to interventional radiology Adjunct to expectant management	[5,36]
Segmental resection (one-step conservative surgery)	Surgical (uterus-preserving)	Variable; favorable in selected cases	Localized invasion No parametrial extension Experienced surgical team	[39,52]
Triple-P procedure	Surgical, multidisciplinary	High uterine preservation in expert centers	Identifiable placental upper margin Preoperative devascularization possible Access to IR and surgical expertise	[36,52]
Methotrexate therapy	Pharmacologic	Inconsistent; no proven benefit	Stable patient No active bleeding Close monitoring possible	[39,51]
Combined approaches (e.g., UAE + MTX)	Multimodal	Case-dependent	Selected patients only High institutional expertise Intensive follow-up	[52,53]

Reported success rates refer to uterine preservation without immediate hysterectomy and survival to hospital discharge; definitions vary across studies and long-term outcomes are heterogeneous.

## Data Availability

Data sharing is not applicable to this article as no new data were created or analyzed in this study. All information discussed is derived from previously published literature.
